# Enhancing short-term crime prediction with human mobility flows and deep learning architectures

**DOI:** 10.1140/epjds/s13688-022-00366-2

**Published:** 2022-11-08

**Authors:** Jiahui Wu, Saad Mohammad Abrar, Naman Awasthi, Enrique Frias-Martinez, Vanessa Frias-Martinez

**Affiliations:** 1 University of Maryland, College Park, USA; 2 CAILab, Universidad Camilo Jose Cela, Madrid, Spain

**Keywords:** Short-term Crime Prediction, Human Mobility Flows, Deep Learning

## Abstract

Place-based short-term crime prediction models leverage the spatio-temporal patterns of historical crimes to predict aggregate volumes of crime incidents at specific locations over time. Under the umbrella of the *crime opportunity theory*, that suggests that human mobility can play a role in crime generation, increasing attention has been paid to the predictive power of human mobility in place-based short-term crime models. Researchers have used call detail records (CDR), data from location-based services such as Foursquare or from social media to characterize human mobility; and have shown that mobility metrics, together with historical crime data, can improve short-term crime prediction accuracy. In this paper, we propose to use a publicly available fine-grained human mobility dataset from a location intelligence company to explore the effects of human mobility features on short-term crime prediction. For that purpose, we conduct a comprehensive evaluation across multiple cities with diverse demographic characteristics, different types of crimes and various deep learning models; and we show that adding human mobility flow features to historical crimes can improve the F1 scores for a variety of neural crime prediction models across cities and types of crimes, with improvements ranging from 2% to 7%. Our analysis also shows that some neural architectures can slightly improve the crime prediction performance when compared to non-neural regression models by at most 2%.

## Introduction

Environmental criminology provides theoretical foundations to study crimes from the perspective of places [19, 48]. Places with different urban functions can be viewed as crime attractors and crime generators [7]. Through the lens of place-based crime prediction, we can study the complex relationship between future and historical crimes, the built environment and social interactions. In this paper, we focus on short-term, place-based crime prediction *i.e.,* the identification of places where there is a high probability of crime incidents in the next day or hour. Short-term crime prediction is generally used to better allocate policing resources so as to respond to crimes more efficiently.

*Crime-prediction with historical data only*. Various models have been developed to tackle place-based short-term crime prediction using historical crime data. Kernel density estimation—which was very common in the early efforts of crime prediction—uses the estimated density of historical crimes as a measure of risk for future crime areas in the short term [12]. Epidemiological models have also been used to explain crime; for example, Mohler *et al.* proposed an epidemic-type aftershock sequence model to utilize the near repetition patterns of historical crimes [31], whereby the spatio-temporal patterns of crimes in one location increase the probability of other incidents occurring at nearby locations in the short term [23]. In addition, the recent popularity of deep learning has brought in several deep learning approaches to model the non-linear spatio-temporal patterns of crime in the short term [18, 21, 22, 51].

*Crime prediction with historical and mobility data.* In the past few years, and motivated by *crime opportunity theories*, researchers have explored enhancing these place-based short-term predictive models with human mobility data [24, 38, 40]. *Crime opportunity theories* attempt to explain the occurrence of crimes in terms of human behaviors by looking into how variations in people’s routine activities might affect the *opportunities* for crime, *e.g.,* the higher the presence of individuals at a given place, the more or less crimes could happen, depending on the type of crime [33]. Place-based short-term crime prediction models have incorporated the crime opportunity theory by modeling routine activities using human mobility data; and have shown that incorporating mobility data can improve the accuracy of the predictions [24, 38, 40]. The mobility data used to predict crime has been extracted from call detail records (CDR) [3], from location-based services such as Foursquare [24, 38], or from social media such as geo-localized Twitter [52]; and has been generally used to compute the *footfall i.e.,* number of people present at a given place. Although footfall has been shown to improve crime prediction accuracy when compared to using only historical crime data [38], recent work by Kadar *et al.* has revealed that incorporating more nuanced mobility data, such as incoming and outgoing flows to/from regions, or regions visited during a trip (a.k.a pass-through flows), can improve the crime prediction accuracy even further [24].

*Limitations.* Kadar *et al.* work has been pivotal—and unique—in showing that more complex mobility features can be used to improve place-based short-term crime prediction models. However, there are three important limitations in that work. First, Kadar’s work computes mobility flows from Foursquare data by using consecutive check-ins to define incoming and outgoing flows. Nevertheless, these flows might represent incomplete mobility behaviors since people might not check-in on Foursquare all the locations visited. Second, to identify regions visited in a trip (pass-through flows), Kadar *et al.* simulate trajectories in a city via shortest-paths routes, since Foursquare does not collect any route (trajectory) information. However, prior research has shown that people do not always make shortest-path decisions when traveling [54]. Third, Kadar’s work only explores non-neural models as predictors. However, extensive recent literature has shown that deep learning approaches can outperform simpler predictive approaches in the context of short-term crime prediction due to their ability to handle complex spatio-temporal data [18, 21, 51].

We posit that although Kadar’s work is an important first effort in the exploration of more complex mobility features as crime predictors, their approach can be improved (i) by using features extracted from *actual* mobility flows, rather than approximating flows from Foursquare data—we propose to compute mobility flows from GPS data collected by location intelligence companies; and (2) by exploring the performance of more complex deep learning models. The main contributions of this paper are:

- An analysis of the effect of mobility features—modeled as flows and computed using GPS data from a location intelligence company—on the accuracy of place-based short-term crime prediction models, when compared to crime predictors solely based on historical crime data. Our results show that mobility features, represented as a comprehensive set of flow metrics between census tracts [26], do in fact enhance the performance of next-day crime prediction models; thus confirming prior work performed with potentially incomplete and simulated flows from Foursquare [24].
- An extensive experimental evaluation of the performance of deep learning predictive models compared to simpler regression models. Our work shows that deep learning models can outperform simpler regression approaches, although the improvement is limited to a maximum 2% increase in the F1 score.
- An extensive experimental evaluation by looking at place-based short-term crime prediction for four cities in the US with diverse demographic characteristics: Baltimore, Minneapolis, Austin and Chicago; and for eight different types of crime divided into two groups: (i) property crimes including arson, burglary, larceny-theft, and motor vehicle theft; and (ii) violent crimes including aggravated assault, forcible rape, murder, and robbery. Crime patterns might differ across geographies and types of crimes; by exploring predictive models across a broad spectrum—the largest to date—we will be able to discuss performance across a large number of settings.

The rest of the paper is organized as follows. Section 2 presents related work, followed by a thorough description of all the datasets used in this paper in Sect. 3. Section 4 presents a description of the short-term crime prediction models we propose while Sect. 5 describes the evaluation results. Finally, Sect. 6 presents the limitations of our approach, followed by conclusions in Sect. 7.

## Related work

We first describe approaches to spatio-temporal modeling and prediction of crime incidents solely based on the use of historical crime data; and we continue with a discussion of prior work showing that incorporating mobility data can enhance crime prediction methods.

### Crime prediction

Crime prediction has been a long standing research topic of interest to researchers from different backgrounds. Environmental criminology has revealed numerous spatio-temporal patterns across different types of crimes [15, 20]. For example, researchers have found that crimes are highly concentrated in space and cluster at a range of spatial scales, with at least half of the crimes taking place in only approximately 5% of street segments in several cities [48]. Over short time ranges, near repeat victimization has been observed in different types of crimes over the world [23] *i.e.,* when a crime incident occurs at one location, there is a temporary increase in the probability that other crime incidents will occur nearby. Over long periods, the concentration of crimes has also been found to be stable: based on the 14 years (1989-2002) of crime reports in Seattle, Weisburd *et al.* revealed that the vast majority of street segments showed a remarkably stable pattern of crime [47].

That crimes stably cluster in both space and time is the basis of crime prediction using historical crimes [36]. In the early efforts of crime prediction, Geographical Information System (GIS) enabled the generation of crime maps that assigned predictive risk scores to places, using techniques such as kernel density estimation based on historical crimes [6, 12]. Mohler *et al.* modeled the near repeat victimization with an epidemic-type aftershock sequence model and conducted randomized controlled field trials with the Los Angeles Police Department (LAPD) [31]; and Mondal *et al.* used space-time permutation models to identify statistically significant crime clusters in Pune (India) [32]. The proliferation of machine learning techniques have further helped modeling the complex and non-linear spatio-temporal correlation of crime incidents as well as other related data sources, such as point-of-interests and 311 urban service requests data [22, 25]. Lately, the research field has been dominated by deep learning architectures that have shown accuracy improvements over simpler approaches, possibly due to its ability to model complex spatio-temporal trends. For example, Duan *et al.* proposed a pure convolution architecture for crime prediction [18]; while other deep learning components, such as recurrent neural network and self-attention have also utilized to jointly model spatio-temporal patterns of crimes [21, 51]. In this paper, we will evaluate the use of deep learning methods for place-based crime prediction models that incorporate mobility data reflecting aggregate spatio-temporal flows across census tracts.

### Modeling crime with human mobility

In addition to the inherent spatio-temporal patterns of crime incidents, there exist various theories about the relationship between human mobility and crime incidents; and Browning *et al.* provide a systematic review for the theoretical foundations at the intersection of place, neighborhood, crimes and human mobility. For example, the *routine activities* theory puts an emphasis on mobility and the social characteristics of micro-places; the *social disorganization* theory has an implicit focus on mobility through the lens of neighborhood-level social interaction [9]; while the *opportunity makes the thief* theory claims that the opportunity is the cause of crime [14] *i.e.,* the higher the presence of *targets* such as people and property, the more crimes could happen.

With the availability of large scale human mobility datasets, such as check-ins, call detail records and GPS data, various studies have provided empirical evidence about the relationship between crime and human mobility for both short- and long-term crime prediction. One of the most common mobility features used in these studies is *footfall*, defined as the number of individuals present in a given area at a given time span. Using footfall and other features, Bogomolov *et al.* built long-term predictive models that could determine crime occurrence in the following month [3]; and Caminha *et al.* showed that increased footfall in a particular area of the city was proportional to the increasing rate of property crimes happening in the region [10]. Kadar and Pletikosa extracted footfall from check-ins, subway and taxi data, along with other census and POI features, to predict the number of crimes for a given census tract in the next year [25]; De Nedai *et al.* proposed a spatially filtered Bayesian Negative Binomial model to study how social, built environment and footfall influence criminal activity [16]; Rumi *et al.* proposed a set of footfall *dynamic* features computed from Foursquare check-ins including visitor count, visitor entropy and homegeneity or region popularity, and showed that these features improved the performance of short-term crime prediction for certain types of crime with F1-score increases of up to 2% [38]; and Stec *et al.* showed that deep learning architectures that use footfall from public transit, together with weather conditions that have been reported to affect crime, enhance the accuracy of crime predictions [40]. In addition to footfall, Wu *et al.* quantified urban spatial structure using human mobility to predict number of crimes for municipalities in the next year [49, 50].

All these works have shown that footfall metrics can improve crime prediction performance when compared to using only historical crime data. However, recent work by Kadar *et al.* has revealed that incorporating more nuanced mobility metrics can improve the prediction performance further [24]. Specifically, Kadar *et al.* used two mobility features extracted from Foursquare data: flows between origin and destination census tracts and pass-thorough flows as regions visited during a trip. While flows measure volume of trips between origin and destination regions, pass-through flows measure regions that were *visited* by travelers without necessarily stopping by. However, the computation of these two metrics was not straight forward given the nature of Foursquare data. First, the flows computed from Foursquare—by creating a flow between two consecutive check-ins—might not reflect all flows between regions since people might choose not to check-in at specific locations thus providing incomplete snapshots of their origin and destination flows. Second, pass-through flows are not available in Foursquare data and as a result, Kadar *et al.* proposed to simulate trajectories via shortest path routes which might or might not reflect the actual routes followed by individuals since shortest paths are not necessarily the way individuals choose their routes, as shown in prior work [54]. To overcome these two limitations, in this paper we propose to build on Kadar’s *et al.* work and explore the use of mobility features extracted from a location intelligence company that can compute actual, complete mobility flows between regions. Using these mobility features, our objective will be to explore whether place-based short-term crime prediction models can be improved when compared to models exclusively based on historical crime data. In addition, Kadar’s work only explored non-neural models as predictors. However, as described in the previous section, extensive recent literature has shown that deep learning approaches can outperform simpler predictive approaches in the context of short-term crime prediction due to their ability to handle complex spatio-temporal data [18, 21, 51]. Thus, our objectives in this paper are twofold: evaluate the effectiveness of using actual mobility flow data in short-term crime prediction models, and analyze the impact of using deep learning predictive approaches.

## Data

We use two types of data: crime incidents and human mobility. In this section, we describe the datasets and provide general statistics for the four cities we evaluate our approach on: Baltimore (Bal), Minneapolis (Min), Austin (Aus) and Chicago (Chi). These four cities were chosen based on the diversity of their demographics, as shown in Table 1, with Baltimore having majority Black and African-American population, Minneapolis majority White, Austin has a high White and Latino and Hispanic population and Chicago with a balanced mix of White, Black and African-American and Hispanic and Latino communities. By replicating the short-term crime prediction analysis across these four cities, we will provide a robust analysis across geographies.

**Table 1:** The percentage of population across race and ethnicity for the four cities according to the American Community Survey (2019 ACS 5-year estimates) [42]. The cities are: Baltimore (Bal), Minneapolis (Min), Austin (Aus) and Chicago (Chi)

	% Not Hispanic or Latino, White Alone	% Black or African-American	% Hispanic or Latino	% Asian
Bal	27.54%	62.46%	5.12%	2.59%
Min	59.80%	19.36%	9.58%	6.13%
Aus	49.08%	7.60%	33.64%	7.34%
Chi	33.61%	29.48%	28.89%	6.40%

### Crime incident data

We obtained the crime incident datasets for the four cities from their open data portals, covering crimes from January to December, 2020.^1^ Each crime incident is associated with the crime category it belongs to and with the time and location where it took place. Crime locations are generally geo-coded to the closest street or block in the city, however, to account for the potential spatial precision inaccuracy, we use a 50-meter buffer to associate crime incidents to urban census tracts (a similar approach has been implemented in prior work *e.g.,* De Nadai et al. [16], Kadar and Pletikosa [25]). Although crime incidents could be associated to smaller spatial units, our choice is determined by the availability of human mobility data at the census tract level only. We group the crime incidents into two types: property and violent crimes, and we will evaluate short-term crime prediction for each type separately. Property crimes include arson, burglary, larceny-theft, and motor vehicle theft; while violent crimes include aggravated assault, forcible rape, murder, and robbery. Table 2 shows the monthly crime density for each city throughout 2020, where monthly crime density is computed as the percentage of census tracts with crime incidents during that month. The table shows that the four cities selected generally suffer from higher volumes of property crimes than violent crimes; and that they represent a diverse group with some cities suffering from higher volumes of violent and property crimes than others.

**Table 2:** Crime occurrence monthly density for the four cities in 2020: Baltimore (Bal), Minneapolis (Min), Austin (Aus) and Chicago (Chi)

		Jan	Feb	Mar	Apr	May	Jun	Jul	Aug	Sep	Oct	Nov	Dec
Property Crime	Bal	28.0%	27.2%	24.6%	22.4%	23.6%	25.0%	24.0%	22.7%	24.7%	25.6%	24.2%	21.3%
	Min	35.0%	33.4%	34.1%	35.3%	37.6%	34.7%	41.6%	43.3%	40.7%	41.3%	37.0%	33.2%
	Aus	32.9%	31.9%	30.6%	30.5%	31.2%	31.5%	31.8%	34.3%	35.0%	33.3%	36.1%	34.4%
	Chi	23.5%	22.6%	19.7%	16.6%	19.6%	20.4%	22.5%	23.5%	22.2%	21.0%	19.7%	18.2%
Violent Crime	Bal	21.6%	21.1%	21.8%	17.0%	21.6%	23.4%	23.2%	23.4%	22.4%	22.5%	21.1%	18.6%
	Min	9.4%	9.3%	10.7%	8.5%	10.3%	13.0%	16.4%	14.6%	13.7%	12.9%	10.4%	8.3%
	Aus	4.0%	3.7%	4.5%	4.2%	5.0%	5.4%	5.7%	5.3%	5.2%	4.7%	5.3%	5.2%
	Chi	11.5%	11.0%	9.9%	8.3%	10.2%	11.6%	12.9%	12.8%	12.4%	11.1%	10.8%	9.3%

### Human mobility data

The pervasive presence of ubiquitous technologies such as smart phones, has allowed for the collection of large-scale human mobility data. Location intelligence companies like SafeGraph, collect pseudonymized mobile GPS location data using SDKs installed on individuals’ mobile phones via mobile apps. SafeGraph offers multiple datasets. For this paper, we have used daily origin-to-destination flows at the census tract (CT) level from January to December, 2020. This dataset is publicly available (see [26]). To extract this dataset, SafeGraph assigns to each device a home location at the census block group level based on its night-time activity. Then, it tracks for each device all the trips from its home location to points-of-interest (POIs) in SafeGraphs’ large POI database. Origin-destination (OD) flows are finally computed by transforming all the home-to-POIs trips to CT(O)-CT(D) trips and by computing the number of devices associated to each OD across all census tracts in a city. OD flow volumes are computed at a daily granularity. Since the devices in SafeGraph’s database account for about 10% of the entire population in the U.S., the OD flow volumes are re-scaled by the census population. It is important to clarify that, unlike the only prior work looking into using flows to predict short-term crimes [24], we use actual origin-destination flows based on GPS data collected by SafeGraph thus enhancing the state of the art.

Table 3 shows general OD flow volume statistics for the four cities under study for the year 2020. For each measure, the table shows the mean and standard deviation of its daily average values across all census tracts in each city. In-city OD flows refer to flows whose origin and destination census tracts (CT(O) and CT(D)) are within the city; while out-of-city OD flows are flows in which either the origin or the destination census tract is outside the city under study. To characterize mobility diversity, the table also shows the number of unique census tracts connected by in-city OD flows and the number of unique counties and states connected by out-of-city OD flows. We can observe that most of the OD flows identified take place within the cities under study, with smaller volumes being associated to trips to counties outside the city, and even a smaller number to trips to other states. Consequently, there is a higher diversity in the number of distinct areas visited inside than outside the city (counties or states). A more detailed description of the features extracted from this dataset is covered in the next section.

**Table 3:** Human mobility flow statistics for the four cities under study: Baltimore (Bal), Minneapolis (Min), Austin (Aus) and Chicago (Chi). The numbers in each cell represent the mean (standard deviation) of the daily average across all census tracts in a given city in 2020. OD flows outside the city are flows that either start or end in a census tract that is not part of the city of interest

	Bal	Min	Aus	Chi
Number of census tracts	200	116	204	809
Volume of in-city OD flow	4040.1 (1733.9)	4004.3 (1653.7)	8167.2 (3866.3)	5307.3 (2821.6)
Volume of out-of-city OD flow	1413.6 (1149.9)	2055.8 (1749.5)	2102.6 (1651.3)	1198.9 (1646.3)
The number of unique census tracts connected by in-city OD flow	38.7 (14.6)	30.5 (10.7)	66.8 (20.2)	61.0 (28.5)
The number of unique counties connected by out-of-city OD flow	14.5 (11.9)	23.6 (20.8)	29.6 (17.2)	15.1 (20.5)
The number of unique states connected by out-of-city OD flow	5.9 (3.3)	7.0 (4.2)	7.7 (4.0)	6.2 (4.0)

## Short-term crime prediction with human mobility flows

As stated in the Introduction, our objective is to analyze the effect of mobility features—modeled as flows and computed using GPS data from a location intelligence company—on the accuracy of place-based short-term crime prediction models implemented with deep learning, when compared to crime predictors solely based on historical crime data, and implemented with simpler, non-neural approaches. In this section, we describe the problem setting for short-term crime prediction with mobility data, present the models we will use in our analysis and describe the experimental and evaluation protocols.

### Problem setting

In this study, we focus on placed-based short-term crime prediction for a given city. For that purpose, a city is divided into *N* spatial units $\mathbf{S}=\{s_{1}, s_{2},\ldots, s_{N}\}$ which for this paper are defined as census tracts. We choose census tracts as spatial units because the human mobility flow dataset is only available at the census tract level. We frame short-term crime prediction as determining whether there will be at least one crime the next day at a given census tract using prior crime and mobility data for that tract. Crime occurrences at a census tract $s_{i}$ on day *t* are denoted as $y_{i,t}$ and $y_{i,t}=1$ is referred to as a crime hotspot.

For each census tract $s_{i}$, we compute two sets of daily predictive features: 1) historical crimes (*C*), defined as the daily number of past crime incidents; the input sequence for crime prediction at day *t* is represented as $\mathbf{C}_{i,t}=\{c_{i,t-T}, c_{i, t-T+1},\ldots, c_{i, t-1}\}$ with *T* being the length of the *look-back* period *i.e.,* the time range used to characterize *history* and $c_{i, t-d}$ being the number of crime incidents *d* days before day *t*; and 2) mobility features (*M*), defined as a set of ten daily features extracted from SafeGraph’s daily OD matrices and denoted as $\mathbf{M}_{i,t} = \{\mathbf{M}_{i,t}^{j} | j \in \{1, 2,\ldots, 10\} \}$ and $\mathbf{M}_{i,t}^{j} = \{m_{i,t-T}^{j}, m_{i, t-T+1}^{j},\ldots, m_{i, t-1}^{j} \}$, where $m_{i,t-d}^{j}$ is the value of the *j*-th mobility feature at *d* days before day *t*. The ten features identified characterize mobility volumes and mobility diversity. Mobility volume features characterize the daily total number of people going in (inflow) and out (outflow) of a census tract within or outside the city under study, which have been shown to be related with the volumes of crime incidents [3, 25, 49]; while mobility diversity features characterize the regional influence, *i.e.*, the number of unique regions visited by in/outflows, including census tracts, counties and states. Past research has shown that crimes committed by visitors are associated to different patterns (behaviors) than those of residents [4]; and that pass-through traffic information improves crime prediction accuracy [24]. Therefore, we extract mobility diversity features to reflect the connections between the census tract $s_{i}$ and other regions. Table 4 shows a summary of all the features used in the short-term crime prediction models. Besides crime and human mobility data, we also add *Day of week* to the feature set to capture the difference between crime data and human mobility behaviors during weekdays and weekends.

**Table 4:** Complete list of predictive (input) features for short-term crime prediction models. For census tract $s_{i}$, inflow (outflow) means $s_{i}$ is the destination (origin) of the OD flow

Types	Features
Crimes	Daily number of crimes
Mobility	Volumes of in-city inflow
Volumes	Volumes of in-city outflow
	Volumes of out-of-city inflow
	Volumes of out-of-city outflow
Mobility	Number of CT connected by in-city inflow
Diversity	Number of CT connected by in-city outflow
	Number of counties connected by out-of-city inflow
	Number of counties connected by out-of-city outflow
	Number of states connected by out-of-city inflow
	Number of states connected by out-of-city outflow
day of week	Day of week

In order to evaluate the effects of predicting short-term crime with the mobility features described, we consider 3 combinations of input (predictive) features to the model: 1) *C*: the input contains only the historical crime features; 2) *M*: the input contains only the mobility features; 3) $C+M$: the input contains both historical crimes and mobility features.

**Figure 1:**
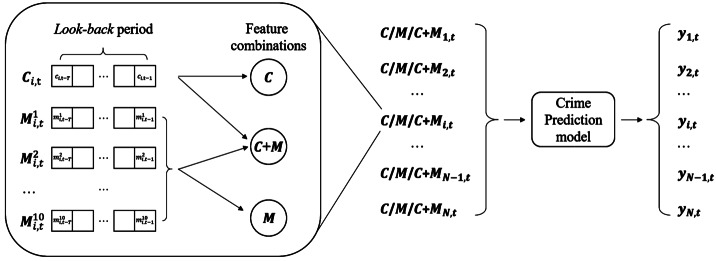
Framework of the place-based short-term crime prediction

*Problem Statement*. Given the temporal sequences of input features (*C*, *M* or $C+M$) within the *look-back* period *T* for all census tracts in a city, predict whether a census tract will be a hotspot in the next day $y_{i,t}=1, i\in [1,N]$. The framework of the problem setting is shown in Fig. 1.

### Models

We explore a wide variety of state-of-art deep learning models to analyze their predictive power when using crime and/or mobility data as input features.

**Figure 2:**
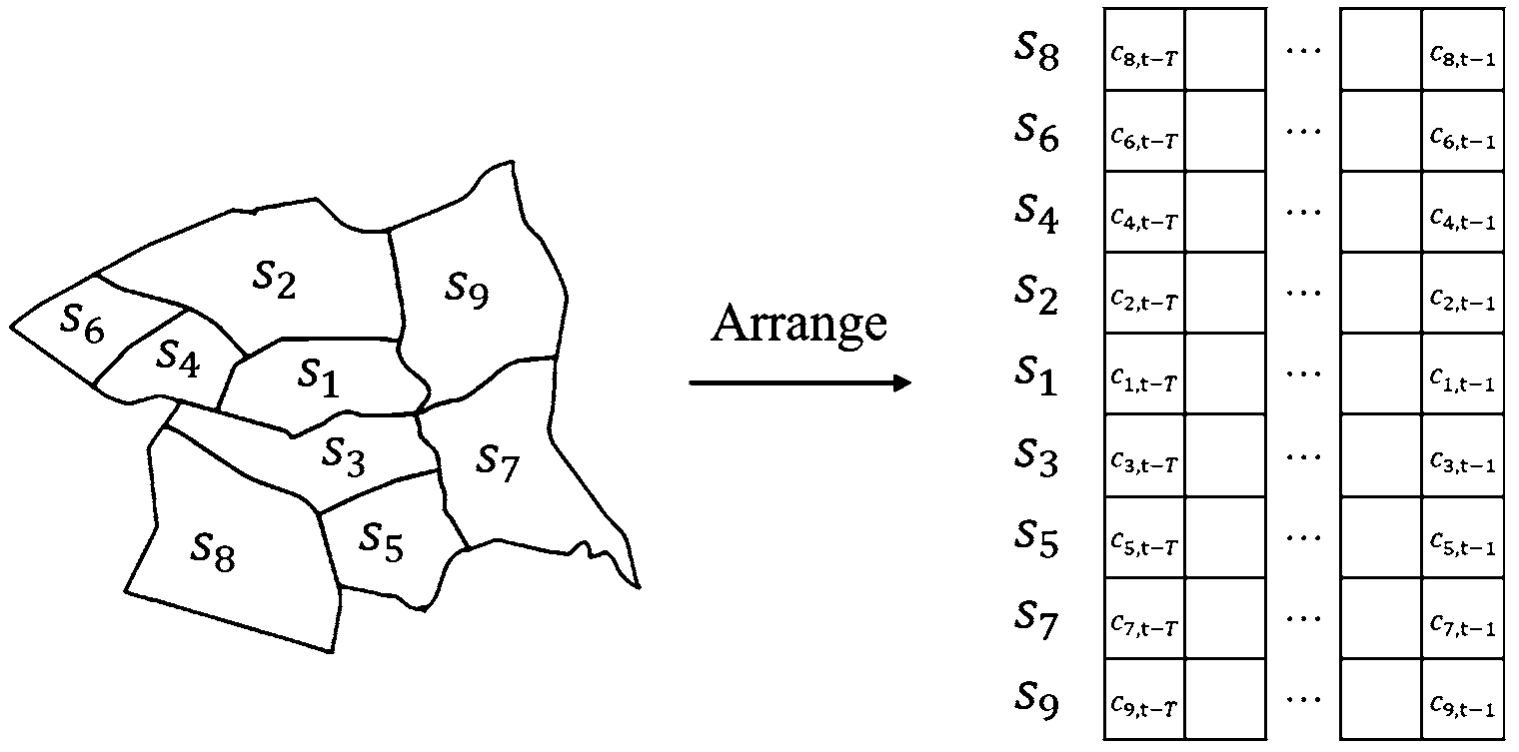
Arrange the nearest neighbors set for the target census tract $s_{1}$ and construct the 2D feature map for historical crimes. In the neighboring set of $s_{1}$, $s_{2}$ and $s_{3}$ is the closest to $s_{1}$; $s_{4}$ and $s_{5}$ are the next closest to $s_{2}$ and $s_{3}$ respectively; $s_{6}$ and $s_{7}$ are the next closest to $s_{4}$ and $s_{5}$; $s_{8}$ and $s_{9}$ are the next closest to $s_{6}$ and $s_{7}$. Similar process is applied to each of the ten mobility features

- *Historical average logistics regression (HALR)*. Historical average is a common baseline in crime prediction studies [12, 18]. It predicts the risk score of a spatial unit being a crime hotspot based on the average number of historical crimes for that unit. To incorporate mobility features within this baseline, we add a logistic regression model. The input of the logistic model are $\overline{\mathbf{C}}_{i,t}$ and $\overline{\mathbf{M}}_{i,t}$, which represent the average of historical crimes and mobility features in the *look-back* period.
- *Gated recurrent units (GRU)*. GRU is a variant of recurrent neural networks and is commonly used for modeling sequential data. In this study, multiple layers of GRU are stacked to model the temporal dependency between the probability of being the next-day crime hotspot $y_{i,t}$ and the input temporal feature sequences $\mathbf{C}_{i,t}$ and $\mathbf{M}_{i,t}$ in the *look-back* sequence for census tract $s_{i}$.
- *Attention crime prediction (Attn)*. Since the success of the Transformer model in natural language processing [45], the attention mechanism has become very popular in modeling sequential data. Here, we use the encoder of the Transformer model with an approach similar to the BERT training setting [17] *i.e.,* we add a *cls* token at the start of the input feature sequences $\mathbf{C}_{i,t}$ and $\mathbf{M}_{i,t}$ in the *look-back* period to predict the probability of crime incidents occurring in census tract $s_{i}$ in the next day.
- *Graph convolution network (GCN)*. By treating all census tracts in a city as nodes in a graph, we can apply graph neural networks to model the spatial dependency of the historical crimes and mobility features among census tracts. In the graph of census tracts, the edges between each pair of census tracts is defined as queen neighbouring (there is an undirected edge between two census tracts if they are queen neighbours, *i.e.*, their boundaries intersect with each other). Graph convolution network (GCN) is one of the earliest neural network architectures for graph structured data [27]. Although more sophisticated graph neural network architectures have been proposed, a simple GCN has been shown to outperform more sophisticated ones if the same hyper-parameter selection and training procedures are used [39]. Therefore, in this study, we adopt GCN for its simplicity and effectiveness for our crime prediction task.
- *GCN with gated 1D convolution (GGConv)*. The above deep learning models consider either the temporal or the spatial dependency of the input features for the census tracts. To model the temporal and spatial dependency simultaneously, Yu *et al.* proposed a spatio-temporal convolutional block, which consists of a two gated 1d convolution for the temporal dependency and one GCN layer for the spatial dependency [53]. For this model, we use the same definition of census tracts graph as for the GCN previously described.
- *Neighbor convolution (NbConv)*. Neighbor convolution models that account for spatio-temporal dependency have been used for crime prediction using historical data over a spatial grid [18]. To adapt this model to our setting, where the spatial units are census tracts (non-regular division), we extract a fixed-length nearest neighbors set for each census tract for which the model outputs the next-day crime prediction. Specifically, we focus on the eight nearest census tracts for each target census tract. We arrange the target census tract in the middle and sort the nearest neighboring census tracts from closest to furthest to form a 2D feature map per input feature, as explained in Fig. 2. Such arrangement allows the kernel of the convolutional layer to model the spatio-temporal dependency through its local receptive field. These 2D feature maps are then input to the full convolution architecture. The original model in [18] contains inception and fractal blocks. In our setting, we discuss results for a model with only the first regular convolution blocks because it provided better performance than the full model.

To sum up, HALR is the baseline model that will be used to compare against all the other deep learning approaches. GRU and Attn will be used to test the importance of modeling the temporal dependency of the input features within each census tract, while GCN models will assess the effect of spatial dependencies among neighboring census tracts on short-term crime prediction. Finally, GGConv and NbConv model both the temporal and spatial dependencies of the input features simultaneously, and we will explore whether using such approach is beneficial to improving short-term crime prediction performance when compared to simpler models.

### Experiment and evaluation protocols

Next, we introduce the experiment and evaluation protocols to evaluate the performance of short-term crime prediction models with mobility features. Given that we have 1 year of data, we chronologically split the dataset into training (6.5 months), validation (0.5 month), and testing (1 month) sets. The validation set is used to tune the learning rate and early stopping *i.e.,* deciding the maximum number of epochs for training. Then we re-train the model using the combination of training and validation set (a total of 7 months) and use the testing set to make next-day predictions (5 months). The overall performance of a model is represented by its monthly F1 score, computed comparing the next-day crime prediction with the daily ground truth over all days for each testing month. This experimental protocol with time series data has also been followed in other related work such as Huang et al. [21].

In order evaluate whether mobility flow features improve short-term crime prediction models, we explore three input feature combinations: 1) Historical crime features only (*C*); 2) Mobility features only (*M*) and 3) Historical crimes and Mobility features ($C+M$). We use the relative change in the F1 score to evaluate the effect of adding mobility features to the short-term crime prediction problem. The F1 score using *C* serves as baseline and the relative change in the F1 score using $C+M$ (*M*) is computed as: $(\frac{F1_{C+M(M)}}{F1_{C}} -1) * 100\%$.

### Model implementation and hyper-parameters

HALR is implemented using its scikit-learn library with the default hyperparameters. All neural network models are implemented with the PyTorch library. The neural networks use Adam as the optimizer with a weight decay of 0.0001 and the learning rate is tuned using the validation set. The dimension of the hidden states for GRU, GCN, GGConv and NbConv is 100. These models have 3 layers of their core blocks *i.e.,* gated recurrent units for GRU, graph convolution for GCN, spatio-temporal block for GGConv and convolution layer for NbConv. The number of nearest census tracts in NbConv is set as 8. As for Attn, we follow the Mini setting of BERT,^2^ where the dimensions of the hidden states are 256, the number of attention heads is 4 and the number of layers of attention is 4. The length of the *look-back* period is set to 14 and an analysis of the sensitivity to this parameter is explained in Sect. 5.2.

## Model performance analysis

**Figure 3:**
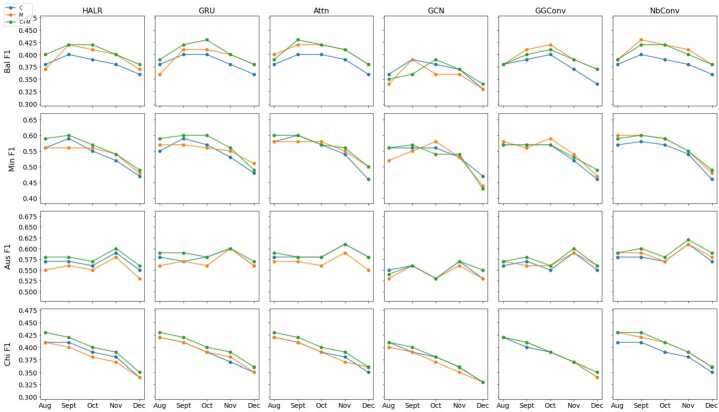
Monthly F1 scores for predicting next-day property crime hotspots. Each row represents the F1 scores for one city across all predictive models: Baltimore (Bal), Minneapolis (Min), Austin (Aus) and Chicago (Chi). The blue lines represent F1 scores for models with only crime data (*C*); the orange lines represent F1 scores for models that use only mobility data (*M*) and the green line are F1 scores both models that use both (*C*+*M*)

Figure 3 shows the monthly F1 scores for predicting property crimes for each model in each city using the three different input combinations: historical crimes only (*C*), mobility features only (*M*) and both (*C*+*M*). In most cases, the F1 scores using *C*+*M* are better than using only *C* or *M*; and this observation is true across cities, test months and models. In other words, adding mobility features—computed using GPS data from location intelligence companies—improves the predictive accuracy of most of the models explored across all cities. A similar trend was observed for violent crimes.

As *C*+*M* is the best combination in most cases, we aim to understand what model is giving the best performance. For that purpose, we calculate the average monthly F1 score across the five test months for each model and city for both property and violent crimes. Tables 5 and 6 shows the results. Overall, GRU, Attn, GGConv, and NbConv have comparable prediction performance and NbConv is the model with best performance in most scenarios, *i.e.*, with the largest F1 scores in three out of four cities for both property crimes and violent crimes. On the other hand, GCN is the model with the worst performance across all scenarios. Since GCN is the only deep learning model in our evaluation that exclusively considers spatial dependency, these results suggest the importance of including temporal dependencies in short-term crime prediction models. We also observe from Fig. 3 that NbConv is the only model that has the better performance using mobility features only (*M*) than using historical crimes (*C*) consistently across different months, cities and types of crimes. Finally, we can also see that the average F1 scores for HALR—the only non-neural architecture we analyze—are lower than those for GRU, Attn and NbConv (up to 2% lower), thus revealing that some deep learning models can in fact provide better results than simpler, non-neural models.

**Table 5:** Average (standard deviation) of monthly F1 score using *C*+*M* for property crime prediction from Aug. to Dec. 2020 for each city

	Bal	Min	Aus	Chi
HALR	0.403 (0.015)	0.557 (0.039)	0.579 (0.014)	0.398 (0.026)
GRU	0.405 (0.017)	0.567 (0.042)	0.586 (0.010)	0.402 (0.027)
Attn	**0.408** (0.018)	0.564 (0.038)	0.588 (0.011)	0.400 (0.024)
GCN	0.363 (0.016)	0.528 (0.051)	0.551 (0.012)	0.375 (0.030)
GGConv	0.391 (0.014)	0.544 (0.033)	0.576 (0.015)	0.386 (0.027)
NbConv	0.407 (0.013)	**0.571** (0.039)	**0.593** (0.012)	**0.406** (0.026)

**Table 6:** Average (standard deviation) of monthly F1 score using *C*+*M* for violent crime prediction from Aug. to Dec. 2020 for each city

	Bal	Min	Aus	Chi
HALR	0.390 (0.024)	0.290 (0.056)	0.159 (0.015)	0.269 (0.023)
GRU	0.393 (0.023)	0.284 (0.054)	**0.166** (0.014)	0.267 (0.021)
Attn	0.398 (0.024)	0.284 (0.055)	0.160 (0.012)	0.266 (0.019)
GCN	0.370 (0.025)	0.275 (0.051)	0.149 (0.008)	0.266 (0.020)
GGConv	0.387 (0.028)	0.285 (0.048)	0.152 (0.005)	0.268 (0.021)
NbConv	**0.400** (0.024)	**0.296**(0.047)	0.159 (0.007)	**0.270** (0.022)

### Measuring the effects of mobility features

To quantify the effect of using mobility features in short-term crime prediction models, we compute the relative change in F1 score between using *C*+*M* or only *M* features and the baseline model with only *C* features, as described in Sect. 4.3. This analysis will measure the effect of using only mobility features or adding mobility features to historical crime features on model performance, when compared to the only crime data baseline. As an example, Table 7 shows the relative change in monthly F1 score using *C*+*M* in Chicago over each test month, from August to December in 2020. We observe that adding mobility features to the models help boost the crime prediction performance in most scenarios (most relative changes are positive for different months and models). However, the improvement of the performance differs across models. We can observe that NbConv makes the best use of mobility features, *i.e.*, the largest relative improvement in F1 scores in all months in Chicago; while the mobility features sometimes hurt the performance of models with a graph convolution layer: GCN and GGConv have a negative relative change in one month.

**Table 7:** Relative change in F1 score using *C*+*M* for property crime prediction in Chicago in each test month

Model	Aug	Sept	Oct	Nov	Dec
HALR	3.7%	3.0%	1.4%	2.3%	2.7%
GRU	4.0%	4.3%	2.5%	3.9%	2.4%
Attn	2.9%	2.2%	2.2%	3.2%	3.7%
GCN	1.2%	1.0%	0.3%	0.1%	−1.8%
GGConv	1.2%	0.3%	0.9%	−0.6%	1.3%
NbConv	**4.3%**	**4.9%**	**5.6%**	**4.2%**	**4.5%**

To be able to analyze the global effect of using mobility features (either *C*+*M* or *M*) across models, cities and types of crimes, we compute the average relative change over the five test months for each model, city and type of crime and discuss main findings. Tables 8 to 11 display the results for all combinations described. Based on these average relative changes, we present the following observations:

**Table 8:** Average relative change in F1 score using *C*+*M* for property crimes over all test months (Aug-Dec) in each city

Model	Bal	Min	Aus	Chi
HALR	**6.1%**	3.6%	1.6%	2.6%
GRU	5.3%	4.6%	1.3%	3.4%
Attn	5.4%	2.7%	0.5%	2.8%
GCN	−0.6%	−1.2%	0.4%	0.2%
GGConv	4.0%	1.4%	**2.4%**	0.6%
NbConv	5.8%	**4.7%**	2.1%	**4.7%**

1) GCN not only has the worst prediction performance but also fails to leverage mobility features, *i.e.,*, the relative changes are mostly negative or small positive values in all cities and types of crimes. In the following observations, we exclude GCN from our analysis.

2) Adding mobility features along with historical crimes as inputs (*C*+*M*) is consistently beneficial to short-term crime prediction for all cities, types of crimes and models, although the extent of improvement varies from 2.4% to 7% increase in F1 scores (see Tables 8 and 10). NbConv achieves the largest improvement in two cities for property crime and in three cities for violent crimes and the second largest improvement in the rest of the cases.

3) Replacing historical crimes input (*C*) with mobility features only (*M*) does not always provide better or comparable crime prediction performance for property crimes (*i.e.*, many relative changes in Table 9 are less than 1%) and often hurts prediction performance for violent crimes (*i.e.*, most relative changes in Table 11 are negative). The exception is NbConv, whose relative changes using *M* are consistently positive and improvements in the F1 scores are often substantial with improvements between 1.4% and 8.2%.

**Table 9:** Average relative change in F1 score using *M* for property crimes over all test months (Aug-Dec) in each city

Model	Bal	Min	Aus	Chi
HALR	3.7%	0.2%	−2.9%	−2.3%
GRU	1.9%	2.4%	−1.6%	0.3%
Attn	4.6%	1.7%	−2.9%	0.4%
GCN	−2.8%	−1.7%	−1.5%	−2.0%
GGConv	4.2%	1.8%	0.9%	0.4%
NbConv	**6.0%**	**2.8%**	**1.4%**	**3.3%**

**Table 10:** Average relative change in F1 score using *C*+*M* for violent crimes over all test months (Aug-Dec) in each city

Model	Bal	Min	Aus	Chi
HALR	4.1%	2.5%	1.7%	**2.8%**
GRU	3.3%	1.8%	4.0%	1.3%
Attn	3.7%	4.5%	1.9%	2.1%
GCN	0.1%	−0.4%	−3.0%	−1.4%
GGConv	1.9%	2.5%	−0.1%	0.7%
NbConv	**5.0%**	**6.6%**	**7.0%**	2.2%

**Table 11:** Average relative change in F1 score using *M* for violent crimes over all test months (Aug-Dec) in each city

Model	Bal	Min	Aus	Chi
HALR	0.4%	−5.7%	−9.2%	−4.0%
GRU	2.3%	−5.4%	−1.3%	−1.8%
Attn	1.1%	−2.8%	−7.7%	−0.7%
GCN	−1.9%	−1.1%	−2.1%	−4.1%
GGConv	2.4%	−1.4%	0.1%	−0.4%
NbConv	**5.2%**	**5.9%**	**8.2%**	**2.4%**

To sum up, these results reveal that using mobility features as predictors of crime, together with historical crime data ($C+M$) or as a substitute for historical crime data (*M*), provides significant improvements in F1 scores across cities and types of crime when the NbConv model is used.

### Effect of length of *look-back* period and length of training months

**Figure 4:**
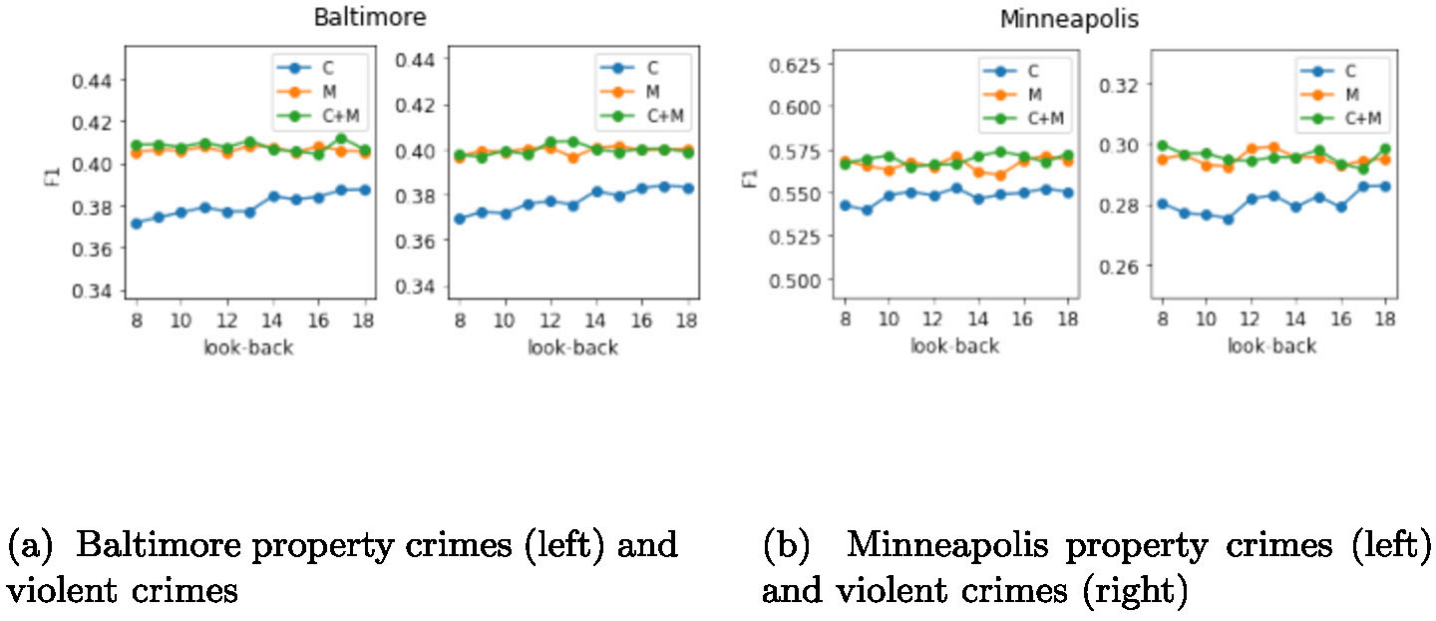
Average F1 score in crimes prediction using NbConv across August to December 2020 with different lengths for the look-back period. Each city has two plots: the one on the left is for property crimes and the one on the right is for violent crimes

**Figure 5:**
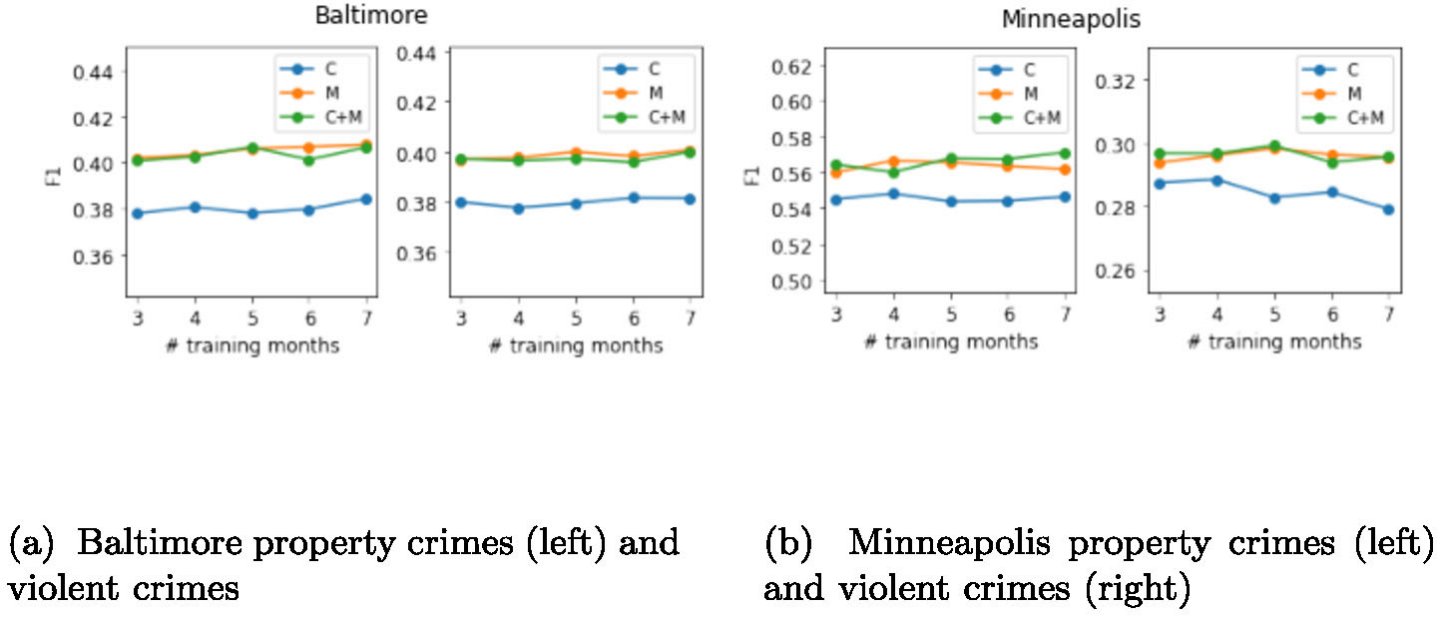
Average F1 score in crimes prediction using NbConv across August to December 2020 with different lengths for the look-back period. Each city has two plots: the one on the left is for property crimes and the one on the right is for violent crimes

In our problem and evaluation setting we have kept two parameters fixed: the length of the *look-back* period is set to 14 and the number of training months (including the validation set) is set to 7. To investigate the effect of these two parameters on our evaluation, we consider a battery of values for the length of the *look-back* and the number of training months, retrain our best performing model—NbConv—and compute the new F1 scores averaged across all testing months for each of the parameter values considered, city, type of crime and combinations of input features (*C*+*M*, *M* and *C*). To test the effect of the *look-back*, we consider values ranging from 8 to 18, with the number of training months fixed to 7. To analyze the effect of the number of months, we consider training months varying from 3 to 7, with *look-back* fixed to 14. The results for Baltimore and Minneapolis are shown in Figs. 4 and 5. The results for Austin and Chicago follow similar trends, and thus are not shown in the paper.

We can observe that the impact of changing the length of the *look-back* period on NbConv models with *M* and *C*+*M* input features is very small, with maximum changes in the F1 score smaller than 1% (see the orange and green lines in the four plots of Fig. 4). On the other hand, the NbConv model with historical crimes only as input features (*C*) is slightly more impacted by changes in the length of the *look-back* period, with F1 scores increasing up to 1.6% as the *look-back* grows until it saturates around *look-back*=14 (value that we select for our analysis). These numbers reveal that the improvements in F1 scores for *C*+*M* and *M* probably represent a lower-bound with potentially larger improvements if the *look-back* period considered was reduced. As for the length of the training months, the impact on F1 scores is also small. We observe maximum F1 score changes of less than 1% and a very slight increase in the F1 score as the length of the training months increases for all input combinations and cities, except for NbConv with input features *C* in Minneapolis. This analysis shows that the F1 scores discussed for NbConv are stable across diverse training lengths.

## Discussion and limitations

Our results have shown that mobility flow features extracted from GPS data collected by location intelligence companies can improve the performance of short-term crime prediction models both for neural and non-neural (HALR) architectures. Interestingly, our analyses have shown that non-neural architectures that use mobility data perform worse than some neural architectures including GRU, Attn and NbConv, but better than others (GCN and GGConv). However, the difference is small, with neural architectures producing short-term crime prediction F1 scores of up to 2% than non-neural approaches.

We have also revealed that mobility flows used together with historical crime data ($C+M$) systematically provide the highest increases in F1 scores when compared to models that only use historical crime data; and that these improvements are pervasive across neural and non-neural models, diverse cities and types of crime. Using only mobility flows (*M*) as crime predictors, instead of historical crime data, also produced systematic improvements in F1 scores across cities and types of crime, albeit only for the NbConv neural model. The F1 score improvements when using mobility flow features—which go from 1.4% to 8.1%—could be potentially lower-bounds of the actual improvements, since our analysis also showed that considering longer look-back periods generally improved the F1 score values. Based on our findings, we propound that mobility features that model flows from GPS data collected by location intelligence companies can be used to improve short-term crime prediction models, and that the NbConv architecture seems to offer an adequate modeling framework to maximize the improvements.

The SafeGraph mobility data that we have used is from 2020. Due to covid-19, 2020 was an abnormal year with the overall mobility heavily reduced specially during the first months of the year [13]. Despite that flattened mobility trend, our results show that mobility features do in fact help improve the crime prediction performance. We posit that our findings provide a lower-bound approximation of the predictive power that mobility features extracted from location intelligence companies can have in place-based, short-term crime prediction models. As mobility goes back to *normal*, or starts to increase defining a new normal, we propound that the predictive power could be potentially higher given that researchers have reported significant relationships between decreasing mobility trends and transmission rates [34]. Similarly, the historical crime data statistics in 2020 were also different than prior years, mostly due to social distancing measures and reduced mobility. Prior work has shown that in 2020, while homicide rates were higher throughout the US when compared to pre-pandemic statistics, robbery and larceny—which potentially requires closer contact—were significantly lower [30]. Despite these anomalies in the crime data, the results presented in this paper are valid and comparable across regions given the changes in crime volumes were similar across the US—including the cities under study in this paper [5].

Predictive policing *i.e.,* the use of predictive models to forecast crime target areas for police intervention and crime prevention [35], comes with its own risks [37]. The historical crime data used in crime prediction models has been shown to suffer from data bias due to under-reporting and under-recording issues [2, 28]. Prior related work has shown that income [15, 41, 46], unemployment rate [29], race [15, 41] or gender [44] are associated to crime under-reporting and under-recording behaviors. For example, white and wealthy crime victims or female-headed household victims in the US are less likely to report to the police [41]; and the police is less likely to record into their databases minor crimes in majority minority-race and immigrant neighborhoods in the US due to the *unworthy victim* perspective [43, 44].

Moving from data to algorithms, researchers have shown that predictive models trained on biased data might reinforce and amplify such bias [8, 28]. For example, Lum and Issac audited *PredPol*, a widely used place-based predictive policing system [31], and found that the locations predicted by the algorithm as crime hotspots were reinforcing data bias. In fact, the authors revealed that the flagged regions were already over-represented in the historical crime data: using *PredPol*, non-white people would be targeted at roughly 1.5 times the rate of whites, in contrast to estimates of drug use by race, which were roughly equivalent across racial classification. Similar bias reinforcement was found for low-income groups who were disproportionately targeted at higher rates.

Beyond data and algorithmic bias arguments, scholars have also raised concerns with respect to the unequal burdens argument *i.e.,* innocent minorities unfairly facing the burden of predictive policing and racial profiling [11]; while others have argued the opposite: the unequal benefits objection states that those burdened are the largest beneficiaries of crime reduction in their communities [1]. In a paradigm shift, recent work puts forward that even if predictive policing significantly reduces crime in minority communities, it can still be unfair and paternalistic, and proposes community-led discussions around predictive policing that will keep communities involved in strategic decision making, including the use (or not) of predictive policing tools in their own communities [37]. We would like to finalize this section by saying that although data bias, algorithmic fairness, and more generally the risks of predictive policing are extremely important issues that we have explored, and continue to explore in our research [49], these are not the focus of this paper.

## Conclusions and future work

In this study, we leverage large-scale human mobility flows for short-term place-based crime prediction implemented with deep learning. The mobility flows are computed from data collected by a location intelligence company and reflect actual, complete population flows between census tracts; thus improving the current state-of-the-art approach that uses flows approximated via Foursquare consecutive visits. To robustly analyze the effect of adding mobility features to next-day crime prediction in terms of prediction accuracy, we conducted comprehensive experiments with a wide range of neural network architectures on cities with diverse demographic characteristics and different types of crimes. Our paper has shown that adding human mobility flow features to historical crimes can improve the F1 scores for a variety of neural short-term crime prediction models across cities and types of crimes. The improvement in F1 scores varies across models. Neighbor convolution architectures (NbConv) that model the spatio-temporal patterns of the input features simultaneously produce the best prediction accuracy when adding mobility features with relative improvements from 2% to 7%. We have also shown that using only mobility flow features—without historical crime data—improves the F1 scores for the NbConv model only, with improvements between 1.4% and 8.2%. Finally, our analysis also shows that some neural architectures can slightly improve the crime prediction performance when compared to regression models by at most 2%. The results discussed in this paper present robust findings confirming that mobility flow data can improve place-based, short-term crime prediction models across diverse geographies and types of crime; and that those improvements can be slightly higher if deep learning approaches are used.

## Data Availability

As described in the paper, crime incident data has been retrieved from the cities’ open data portals via these links: Baltimore: https://data.baltimorecity.gov/; Minneapolis: https://opendata.minneapolismn.gov/; Austin: https://data.austintexas.gov/; and Chicago: https://data.cityofchicago.org. On the other hand, the human mobility flow data is publicly available in: https://github.com/GeoDS/COVID19USFlows. Further dataset details are provided in [26].

## Footnotes

1 Bal: https://data.baltimorecity.gov/; Min: https://opendata.minneapolismn.gov/; Aus: https://data.austintexas.gov/; Chi: https://data.cityofchicago.org/;

2 https://github.com/google-research/bert
